# Functionalized Scaffold and Barrier Membrane with Anti-BMP-2 Monoclonal Antibodies for Alveolar Ridge Preservation in a Canine Model

**DOI:** 10.1155/2020/6153724

**Published:** 2020-09-22

**Authors:** Seiko Min, Taewan Kim, Oksu Kim, Carames Goncalo, Tadahiko Utsunomiya, Takashi Matsumoto, Kayo Kuyama, Nikola Angelov

**Affiliations:** ^1^Department of Periodontics and Dental Hygiene, University of Texas Health Science Center at Houston, Houston, TX, USA; ^2^Department of Periodontics, University of Pennsylvania School of Dental Medicine, Philadelphia, PA, USA; ^3^Department of Periodontology, Dental Research Institute, School of Dentistry, Chonnam National University, Gwangju, Republic of Korea; ^4^Department of Periodontology, Implantology Institute, Lisbon, Portugal; ^5^Department of Pathology, School of Dentistry, Nihon University at Matsudo, Chiba, Japan; ^6^Division of Diagnostic Pathology, Nihon University Hospital School of Dentistry at Matsudo, Chiba, Japan

## Abstract

**Introduction:**

The aim of this study was to investigate the ability of anti-bone morphogenetic protein 2 monoclonal antibody (anti-BMP-2 mAb) to functionalize scaffolds to mediate bone regeneration in a canine model.

**Materials and Methods:**

The mandibular right premolar 4 (PM4) was extracted in eight beagle dogs and grafted with anti-BMP-2 mAb+anorganic bovine bone mineral with 10% collagen (ABBM-C) and porcine bilayer native collagen membrane (CM). The ABBM-C and CM were functionalized with either anti-BMP-2 mAb (test group) or an isotype matched control mAb (control group). Animals were euthanized at 12 weeks for radiographic, histologic, and histomorphometric analyses. Outcomes were compared between groups.

**Results:**

3D imaging using cone beam computed tomography (CBCT) revealed that sites treated with ABBM-C and CM functionalized with anti-BMP-2 mAb exhibited significantly more remaining bone width near the alveolar crest, as well as buccal bone height, compared with control groups. Histologic and histomorphometric analyses demonstrated that in anti-BMP-2 mAb-treated sites, total tissue volume was significantly higher in the coronal part of the alveolar bone crest compared with control sites. In anti-BMP-2 mAb-treated sites, bone formation was observed under the barrier membrane.

**Conclusion:**

Functionalization of the ABBM-C scaffold and CM appeared to have led to bone formation within healing alveolar bone sockets.

## 1. Introduction

Numerous studies have demonstrated that significant bone resorption occurs as an inevitable biological event following tooth extraction without additional intervention [1–11]. The rates of loss of alveolar bone width and height in the first three months following extraction are approximately 0.25 mm and 0.2 mm, respectively [7]. A systematic review concluded that in the first six months, the dimensional changes in the alveolar ridge after tooth extraction lead to a mean horizontal width loss of 3.8 mm and a mean vertical height loss of 1.24 mm [12]. Moreover, spontaneous postextraction healing leads to significant alveolar bone contour loss [13].

The magnitude of bone resorption appears more prominent when the initial thickness of the buccal bone wall is less than 1.0 mm [6]. The prevalence of a thin buccal wall was investigated and a study revealed that most teeth in the anterior maxilla have a thin buccal bone [14].

The subsequent dimensional loss of the alveolar ridge following tooth extraction often results in different complications, including insufficient bone volume for dental implant placement in the optimal position and an esthetic defect. The esthetic complication such as a peri-implant soft tissue deficiency can be caused mostly by implant malposition [15].

To minimize the postextraction alveolar ridge dimensional loss, systematic reviews have recommended socket grafting at the time of extraction for ridge preservation [16, 17]. Various biomaterials used for ridge preservation have been evaluated including autograft [18, 19], allograft [20], xenograft [18, 21–25], and alloplast [24, 26–28]. These graft materials have been protected by different barrier devices, including resorbable membranes [20, 29, 30], nonresorbable membranes [31], autogenous soft tissue plugs [32], and extraction socket devices [7–11].

Tissue engineering strategies that combine osteoconductive scaffolds with osteoinductive mediators, such as recombinant human bone morphogenetic protein 2 (rhBMP-2), have been reported [33–37]. Application of rhBMP-2 has been expanded for bone repair; however, the numbers of reported complications have been increasing [38–48].

As an alternative approach to using rhBMP-2, the application of anti-bone morphogenetic protein 2 monoclonal antibody (anti-BMP-2 mAb) was proposed to capture endogenous BMP-2 and homologous ligands in an approach known as antibody-mediated osseous regeneration (AMOR) [49]. Anti-BMP-2 mAbs are able to mediate differentiation of local progenitor stem cells into osteoblast-like cells, thereby promoting bone repair and regeneration. Anti-BMP-2 mAbs can capture endogenous BMPs that supply the signals for repairing different types of bony defects, such as BMP-2, BMP-4, and BMP-7. The capability of AMOR has been tested in various defect models [49–57].

This exploratory study used an AMOR approach in a canine model to investigate a potential therapeutic intervention for preserving the alveolar ridge following tooth extraction. The anti-BMP-2 mAb was employed to functionalize both a scaffold and a barrier membrane.

## 2. Materials and Methods

### 2.1. Animals

The research protocol of this study was approved by the Institutional Animal Care and Use Committee (IACUC) of the University of Southern California (USC, Los Angeles, CA, USA). Eight beagle dogs (four years old, weighing 10 to 11 kg) were used in this study and maintained on a soft diet with food and water *ad libitum*.

### 2.2. Materials

#### 2.2.1. Antibodies and Scaffold

The experimental mAb was a chimeric anti-BMP-2 mAb with cross-reactivity to BMP-4 and BMP-7. The control mAb was an isotype matched mAb specific for the KLH peptide that had no specific affinity for BMP-2 [54]. A concentration of 25 *μ*g/ml of mAb was chosen based on the results of our previous studies [54]. Anti-BMP-2 mAb and isotype matched control mAb were immobilized on deproteinized anorganic bovine bone mineral with 10% collagen (ABBM-C; Bio-Oss Collagen®, Geistlich, Pharma AG, Wolhusen, Switzerland) as well as porcine bilayer native collagen membrane (CM; Bio-Gide® membrane, Geistlich, Pharma AG, Wolhusen, Switzerland) as previously described [54]. Briefly, the ABBM-C and CM were incubated at room temperature with mAb diluted with phosphate-buffered saline (PBS) for one hour prior to implantation into the sockets after tooth extraction. All antibody preparations were made by two of the coauthors (S.M., O.K.).

#### 2.2.2. Surgical Protocol

**Figure 1:**
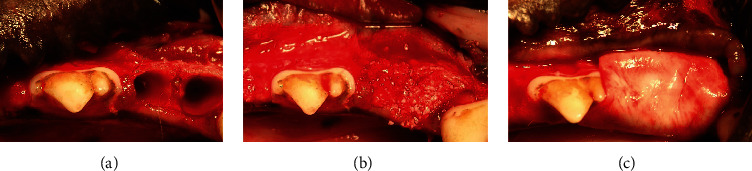
(a) The mandibular right premolar 4 (PM4) was extracted with flap elevation as atraumatically as possible. (b) The sockets were filled with anorganic bovine bone mineral with 10% collagen (ABBM-C) functionalized with either anti-bone morphogenetic protein 2 monoclonal antibody (anti-BMP-2 mAb, test group) or isotype matched control mAb (control group). (c) The sockets filled with ABBM-C were covered by porcine bilayer native collagen membrane (CM) functionalized with either anti-BMP-2 mAb or isotype matched control mAb. The marginal gingiva was then approximated to achieve primary wound closure with a nonresorbable suture.

Preoperatively, animals were given atropine (0.05 mg/kg) and sedated with ketamine (10 mg/kg subcutaneously). Ketamine/xylazine was administered to induce anesthesia. Animals were intubated and ventilated with an isoflurane/oxygen machine. Anesthesia was maintained by isoflurane (1-4%). Animals were draped and then periorally swabbed with cetrimide (1% solution), followed by chlorohexidine gluconate (0.05% solution). Before surgeries, dental prophylaxis was performed and all surgical sites were swabbed with chlorhexidine gluconate (0.12% solution). After intravenous ketamine (5 mg/ml) and intramuscular tiletamine-zolazepam (5-10 mg/kg) were administered, local anesthesia was achieved by using lidocaine HCl (2%) with 1 : 100,000 epinephrine. The mandibular right premolar 4 (PM4) was then extracted with flap elevation as atraumatically as possible in each of the eight dogs (Figure 1(a)). Then, the sockets were filled with ABBM-C functionalized with either anti-BMP-2 mAb or isotype matched control mAb (Figure 1(b)). After the sockets were filled with ABBM-C, they were covered by functionalized CM with either anti-BMP-2 mAb or isotype matched control mAb (Figure 1(c)). The marginal gingiva was then approximated to achieve primary wound closure with nonresorbable polytetrafluoroethylene (PTFE) monofilament suture (4-0 Cytoplast™ suture; Osteogenics, Lubbock, TX, USA). Three of the coauthors (S.M., O.K., and C.G.) performed all surgical procedures. The eight extraction socket sites were then randomly assigned to either of the two experimental treatment groups: (1) test (*N* = 4): anti-BMP-2 mAb+ABBM-C+CM and (2) control (*N* = 4): isotype matched control mAb+ABBM-C+CM.

#### 2.2.3. Postoperative Care

After surgery, the animals were maintained on a soft diet. On alternate days, oral hygiene was performed by applying chlorhexidine gluconate (0.12% solution) with an ultrasoft toothbrush. Postoperative analgesia was administered 2 times per day for 2 days (buprenorphine, 0.05 mg/kg weight), after which the comfort level of the animals was assessed and additional analgesia was provided as needed. Sutures were removed 14 days later. At 12 weeks after tooth extraction, the animals were euthanized so that cone beam computed tomography (CBCT) and histologic and histomorphometric analyses could be conducted. The mandible of each dog was block resected, labeled, and fixed for 10 days in a 10% buffered formalin solution.

#### 2.2.4. CBCT Analysis

**Figure 2:**
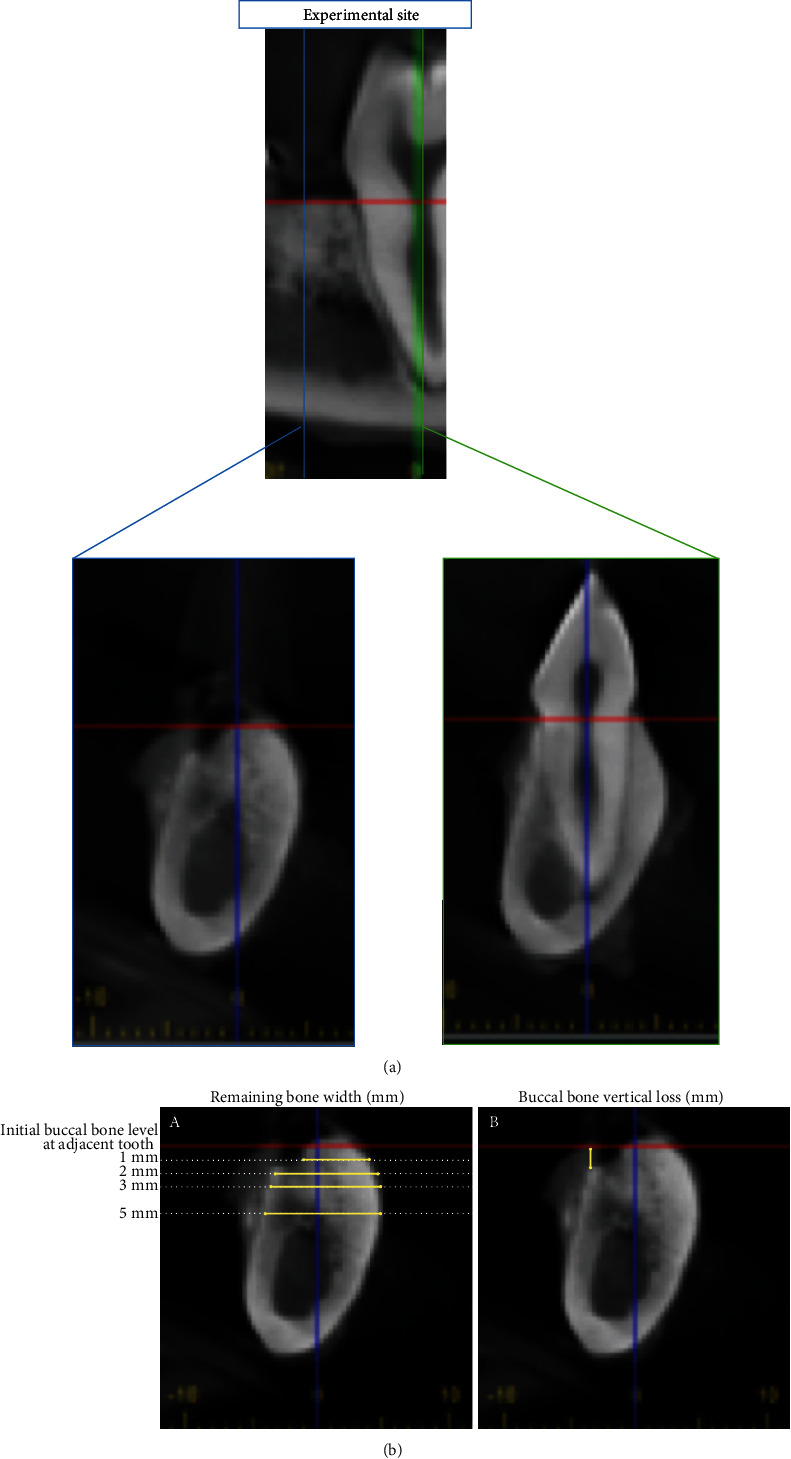
Repeatable anatomical structures such as the adjacent crest level (red line in upper image) and long axis of the adjacent tooth (blue line in lower two images) were used as references to measure dimensional alveolar bone change. (a) Remaining bone width at different levels of 1 mm, 2 mm, 3 mm, and 5 mm from the bone crest at the adjacent tooth and (b) buccal bone height level relative to bone crest at the adjacent tooth were measured at the buccal thirds of the examined alveolus.

Resected mandibles were imaged with CBCT (J. Morita Veraviewepocs® 3D F40, J Morita USA, Irvine, CA, USA), followed by quantitative analysis to measure mineralized tissue detected at defined locations within the grafted sites. Each of the specimens was placed in a sample holder and was scanned using high resolution. After scanning, the acquired data were imported into 3D image analysis software (i-Dixel 2.0 software, J Morita USA, Irvine, CA, USA) for quantitative analysis. A global thresholding procedure was used to segment the bone tissues. Bone tissues within the defects were defined using a threshold equal to -360 HU. The proportion of bone volume occupying the defect virtual space was measured, allowing quantitative comparisons between the test and control groups. CBCT linear measurements were made at 12 weeks postsurgery, as follows: (1) remaining bone width at different levels (1, 2, 3, and 5 mm relative to the bone crest of the adjacent first molar tooth (Figure 2(a)) and (2) buccal bone vertical level relative to the crestal bone of the adjacent first molar (Figure 2(b)). One coauthor (S.M.) performed all CBCT measurements. Repeated measurements were conducted on 10% of the sites selected randomly. The first and second measurements differed by less than 5%, demonstrating intrarater reproducibility of the analysis.

#### 2.2.5. Histologic and Histomorphometric Analyses

**Figure 3:**
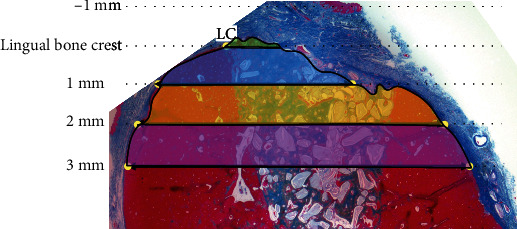
Landmarks for histomorphometric analysis, showing lingual crest (LC) as a relatively stable reference. Additional landmarks relative to the LC are represented at 1 mm coronal (i.e., -1 mm) and 1, 2, and 3 mm apical to the LC. The amounts of total tissue volume in different zones relative to the LC (0-1 mm coronal, 0-1 mm apical, 1-2 mm apical, and 2-3 mm apical) were measured.

Harvested biopsy samples were fixed in 4% paraformaldehyde followed by decalcification in 10% EDTA for 14 days. Excised specimens were embedded in paraffin and then serially sectioned (4 *μ*m thickness) and placed on glass slides. Deparaffinization was performed by immersing in xylene, followed by decreasing ethanol concentrations and washing with water. Azan-Mallory staining was applied to the sections. Images were qualitatively examined under a microscope (CX21^Ⓡ^ Olympus Optical Co., Tokyo, Japan). Histomicrographs were captured with a digital camera and analyzed by using image analysis software (Soft Image System GmbH, Münster, Germany). NIH ImageJ software (U.S. National Institutes of Health, Bethesda, Maryland, USA) was used for histomorphometric analysis to measure the total tissue volume within different areas at 0-1 mm coronal to the lingual bone crest and at 0-1 mm, 1-2 mm, and 2-3 mm apical to the lingual bone crest (Figure 3). One coauthor (T. K.) performed all histomorphometric measurements. Standard methods and nomenclature of the American Society for Bone and Mineral Research (ASBMR) were utilized to define various components of the specimens [58].

#### 2.2.6. Statistical Analysis

The mean and standard deviations were calculated for CBCT and histomorphometric analysis. The Mann–Whitney *U* test was used for pairwise comparisons of the remaining bone width and buccal bone height, and total tissue volume was calculated at different locations. The SPSS software program (IBM SPSS statistics 23, IBM, Armonk, NY, USA) was used for statistical analysis and *P* < 0.05 was considered to be statistically significant.

## 3. Results

### 3.1. Clinical Observations

All surgical sites healed uneventfully with minimal inflammation and no signs of infection.

### 3.2. CBCT Analysis

**Figure 4:**
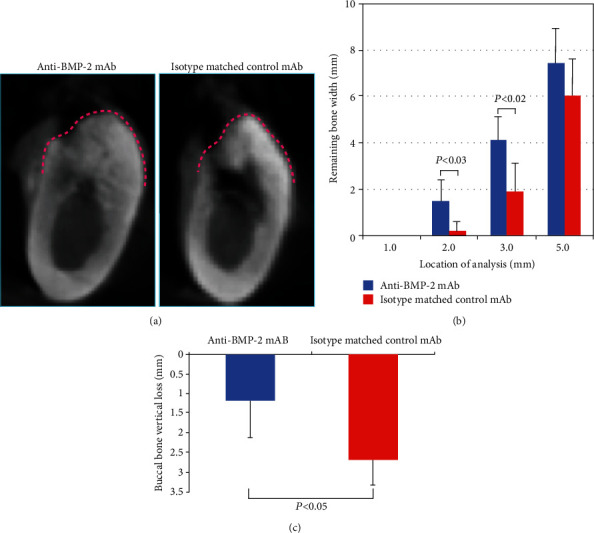
(a) Representative cone bean computed tomography (CBCT) images of anti-bone morphogenetic protein 2 monoclonal antibody- (anti-BMP-2 mAb-) treated site and isotype matched control mAb-treated site. (b) Remaining bone width at 1, 2, 3, and 5 mm from the bone crest at the adjacent tooth: a statistically significant difference in remaining bone width at 2 mm and 3 mm was found between the test group (*N* = 4) and control group (*N* = 4) (*P* = 0.03, *P* = 0.02, respectively). (c) Bone height level at buccal aspect (mm): the amounts of buccal bone height loss in the anti-BMP-2 mAb-treated sites (*N* = 4) and the isotype matched control mAb-treated sites (*N* = 4) were 1.17 ± 0.94 mm and 2.69 ± 0.63 mm, respectively, and were statistically significantly different (*P* = 0.03).

Representative CBCT images illustrate wider alveolar crest in experimental sites treated with scaffold and membrane functionalized with anti-BMP-2, compared with control sites (Figure 4(a)). The alveolar crest also appears to have higher density in the experimental site.

### 3.3. Quantitative Analysis of Bone Width

For anti-BMP-2 mAb, the remaining mean bone widths in test sites at 1, 2, 3, and 5 mm relative to the adjacent crestal bone were 0.0 ± 0.0 mm, 1.5 ± 0.9 mm, 4.1 ± 1.0 mm, and 7.4 ± 1.5 mm, respectively (Figure 4(b), Table 1). Comparatively, the residual mean bone widths of control sites were 0.0 ± 0.0 mm, 0.2 ± 0.4 mm, 1.9 ± 1.2 mm, and 6.0 ± 1.6 mm, respectively. The remaining bone widths at anti-BMP-2-treated sites were statistically significantly higher at 2 and 3 mm compared with control sites (*P* = 0.03, *P* = 0.02, respectively).

### 3.4. Quantitative Analysis of Buccal Bone Height

For anti-BMP-2 mAb, the buccal crest of anti-BMP-2-treated sites was located 1.17 ± 0.94 mm apical to the crestal bone of adjacent teeth. In contrast, the buccal crest of control sites treated with isotype matched control mAb was located 2.69 ± 0.63 mm apical to that of adjacent teeth (Figure 4(c), Table 2). A statistically significant difference was found between anti-BMP-2-treated sites and control sites (*P* = 0.03).

### 3.5. Histologic Observation

**Figure 5:**
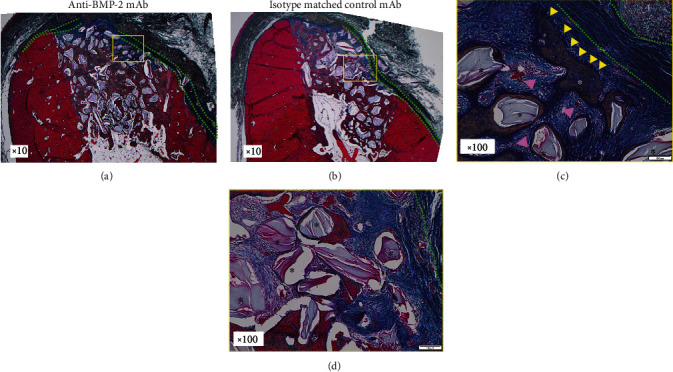
Histology at low magnification in the (a) anti-bone morphogenetic protein 2 monoclonal antibody- (anti-BMP-2 mAb-) treated site and (b) isotype matched control mAb site. Outline of alveolar bone in control sites appeared as a knife-edged shape due to buccal bone loss. The residual barrier collagen membrane (CM) was observed in both test and control sites (green dotted lines). Osteogenesis was observed within the entire extraction socket of both test and control sites. Histology at high magnification in (c) anti-BMP-2 mAb-treated and (d) isotype matched control mAb sites. The micrographs showed osteoblast-like cells (yellow arrowheads) as well as blood vessels (pink arrowheads) participating in active bone formation. The superficial anorganic bovine bone mineral (ABBM) particles (black asterisks in d) in control sites showed fibrotic encapsulation, while osteogenic cells as well as osteoid bone formation around residual ABBM graft particles were noted in test sites (white asterisk in c).

Histologic examination revealed well-defined extraction socket defects with clear demarcation between the woven bone and more mature lamellar bone by Azan-Mallory staining of both anti-BMP-2 mAb-treated sites and control sites (Figures 5(a) and 5(b)). The barrier CM persisted underneath mucosal tissues overlying the extraction orifice of test sites treated with anti-BMP-2 mAb (green dotted lines). In contrast, the CM in control sites appeared to have been significantly more resorbed, accompanied by in-growth of mucosal tissues into the graft. The superficial ABBM particles in control sites appeared to be mostly encapsulated in fibrous tissue (Figures 5(b) and 5(d)). The area underneath the CM in anti-BMP-2 mAb-treated sites was characterized by an abundance of osteoid bone surrounding residual graft particles, as well as vascular tissue (Figures 5(a) and 5(c)). The new bone found within test sites appeared to be characterized by reversal lines, marking the remodeling stage of osteogenesis. In contrast, the bone within control sites was more sparse and consisted of less mature woven bone (Figures 5(b) and 5(d)).

Osteogenesis was observed within the entire extraction socket of both the test and control sites (Figures 5(a) and 5(b)). A polarized pattern of osteogenesis was observed with the most mature bone in the apical region, gradually transitioning to less mature woven bone near the alveolar crest (Figures 5(a) and 5(b)).

### 3.6. Quantitative Histomorphometric Analysis

**Figure 6:**
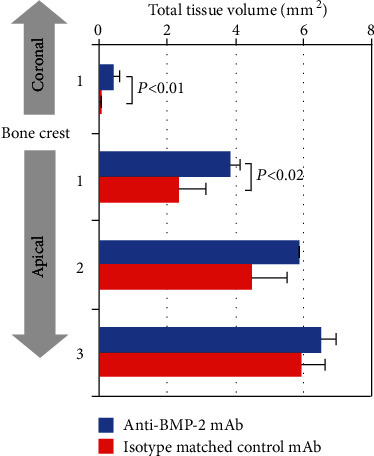
The amounts of total tissue volume within the extraction socket at 0-1 mm coronal to the lingual crest and at 0-1 mm, 1-2 mm, and 2-3 mm apical to the lingual crest. The anti-bone morphogenetic protein 2 monoclonal antibody- (anti-BMP-2 mAb-) treated sites (*N* = 4) revealed a statistically significant higher amount of total tissue volume at 0-1 mm coronal as well as at 0-1 mm apical of the alveolar bone crest relative to the lingual crest compared with the isotype matched control mAb-treated sites (*N* = 4) (*P* = 0.01, *P* = 0.02, respectively). Landmarks used for the analysis are shown in Figure 3.

The landmarks used for quantitative histomorphometric analysis are shown in Figure 3. The results shown in Figure 6 and Table 3 demonstrated that sites treated with scaffold and CM functionalized with anti-BMP-2 (test), but not sites treated with isotype matched control mAb, had bone growth coronal to the alveolar lingual crest (*P* = 0.01). In the zone up to 1 mm apical to the bone crest, there was significantly more bone volume in anti-BMP-2 mAb-treated sites than in control sites (*P* = 0.02).

## 4. Discussion

A variety of graft materials including autogenous [18, 19], xenogenic [18, 21–25], allogenic [20], and alloplastic [24, 26–28] materials have been used for grafting of extraction sockets for ridge preservation. In addition to these traditional grafts, newer scaffolds and biologics developed for tissue engineering have been adopted for ridge preservation [59–62]. These biologics have included growth factors and platelet concentrates [63–65].

One of the most investigated biologics is rhBMP-2, which was approved by the U.S. Food and Drug Administration (FDA) for clinical use to repair bone defects [66–68]. The growing clinical use of rhBMP-2 has been associated with numerous complications such as graft migration [69], formation of neutralizing antibodies against BMP-2 [69], and extreme edema that may obstruct the airway or affect critical structures [46]. Additional disadvantages of exogenous growth factors (e.g., rhBMP-2) include a short biological half-life and lower biologic activity compared to the autogenous analog [70] that necessitates the use of high doses of rhBMP-2 to achieve the therapeutic effect.

To circumvent some of the problems associated with exogenous growth factors, a novel tissue engineering approach for bone regeneration known as AMOR was developed [49]. We previously reported that anti-BMP2-mAb induced osteogenic differentiation *in vitro* and *de novo* bone formation *in vivo*, by using different types of bone defects in animal models that have shown the ability of anti-BMP2-mAb to mediate bone regeneration including rat [49, 50, 52], rabbit [51], canine, and nonhuman primate [56, 57].

The current study is the first to investigate the efficacy of a scaffold and barrier membrane functionalized with anti-BMP-2 mAb for ridge preservation in a canine model.

Following tooth extraction, the anti-BMP-2 mAb immobilized on the ABBM-C was implanted into an extraction socket and was protected by anti-BMP-2 mAb immobilized on a CM.

The present study showed that the anti-BMP-2 mAb-treated sites had statistically significantly greater remaining bone width and buccal bone height, as well as higher total tissue volume, compared with control mAb-treated sites. These favorable outcomes may be attributed to the ability of the anti-BMP-2 mAb to capture endogenous BMP-2, BMP-4, and BMP-7 and increase their *in vivo* persistence [50]. The barrier CM functionalized with anti-BMP-2 mAb was utilized not only for cell occlusion properties and space-making abilities but also for bioactive properties that promote bone regeneration by capturing endogenous BMP-2, BMP-4, and BMP-7.

Radiographic assessment demonstrated that both the buccal bone height level relative to the bone crest at an adjacent tooth and the remaining bone width at 2 and 3 mm relative to the bone crest at an adjacent tooth in the anti-BMP-2 mAb-treated site was statistically significantly higher than that of the control mAb-treated site (*P* = 0.03). Furthermore, histologic observations in the anti-BMP-2 mAb-treated site revealed the presence of bone formation with deposition of active osteogenic cells, including osteoblast-like cells as well as osteoclast-like cells beneath the remaining barrier CM.

One of major findings from this study was that histomorphometric analysis showed a statistically significantly higher total tissue volume at 0-1 mm coronal as well as at 0-1 mm apical to the lingual bone crest in the anti-BMP-2 mAb-treated site compared to that in the control mAb-treated site (*P* < 0.05). The alveolar crest contour in the anti-BMP-2 mAb-treated sites was restored more significantly compared with that in the control mAb-treated sites. Therefore, the current data demonstrated that the use of a scaffold and barrier membrane functionalized with anti-BMP-2 mAb enhanced bone regeneration for ridge preservation. It is also worthwhile to note that in the present study, AMOR showed no signs of a severe inflammation reaction. This finding can possibly be attributed to the low concentration of anti-BMP-2 mAb that is needed to capture endogenous BMP-2 to enhance bone regeneration within the extraction socket.

The present study has a number of limitations, including a small sample size and lack of multiple time points to examine the kinetics of wound healing. We have plans to initiate additional studies with a larger sample size and longer duration to investigate the utility of AMOR for management of complex tooth extractions.

## 5. Conclusion

This study investigated the functionalization of a scaffold and barrier membrane with anti-BMP-2 mAb for extraction socket grafting in a canine model. The application of AMOR for socket grafting was accompanied by increased bone volume and more mature bone formation within the extraction sockets.

## Figures and Tables

**Table 1:** Remaining bone width at 1, 2, 3, and 5 mm relative to the adjacent crestal bone.

Treatment group	Remaining bone width (mm, mean ± SD) at four test sites			
	1 mm	2 mm	3 mm	5 mm
Anti-BMP-2 mAb	0.0 ± 0.0	1.5 ± 0.9^∗^	4.1 ± 1.0^†^	7.4 ± 1.5
Isotype matched control mAb	0.0 ± 0.0	0.2 ± 0.4	1.9 ± 1.2	6.0 ± 1.6

For anti-BMP-2-treated sites vs. control sites: ^∗^*P* = 0.03 and ^†^*P* = 0.02. Anti-BMP-2 mAb: anti-bone morphogenetic protein 2 monoclonal antibody; SD: standard deviation.

**Table 2:** Location of buccal crest relative to crestal bone of the adjacent teeth.

Treatment group	Buccal bone height (mm, mean ± SD)
Anti-BMP-2 mAb	1.17 ± 0.94^∗^
Isotype matched control mAb	2.69 ± 0.63

^∗^ *P* = 0.03 for anti-BMP-2-treated sites vs. control sites. Anti-BMP-2 mAb: anti-bone morphogenetic protein 2 monoclonal antibody.

**Table 3:** Quantitative histomorphometric analysis of sites treated with scaffold and collagen membrane (CM) functionalized with anti-BMP-2 mAb.

Treatment group	Total tissue volume (pixels, mean ± SD)			
	0-1 mm coronal	0-1 mm apical	1-2 mm apical	2-3 mm apical
Anti-BMP-2 mAb	0.45 ± 0.16^∗^	3.85 ± 0.30^†^	5.87 ± 0.03	6.54 ± 0.42
Isotype matched control mAb	0.02 ± 0.05	2.32 ± 0.82	4.48 ± 1.04	5.93 ± 0.72

For anti-BMP-2-treated sites vs. control sites: ^∗^*P* = 0.01 and ^†^*P* = 0.02. Landmarks used for the analysis are shown in Figure 3. Anti-BMP-2 mAb: anti-bone morphogenetic protein 2 monoclonal antibody; SD: standard deviation.

## Data Availability

The data used to support the findings of this study are available from the corresponding author upon request.
